# Ovarian Tissue Culture to Preserve Fertility in Transgender Male Patients after Hormonal Treatment

**DOI:** 10.1055/s-0042-1742410

**Published:** 2022-02-09

**Authors:** Alessandra Leal Bottini, Vânia Marisia Santos Fortes dos Reis, Edison Capp, Ilma Simoni Brum da Silva, Lúcia Maria Kliemann, Helena von Eye Corleta

**Affiliations:** 1 Programa de Pós-Graduação em Ginecologia e Obstetrícia, Universidade Federal do Rio Grande do Sul, Porto Alegre, Brazil; 2 Department of Gynecology and Obstetrics, School of Medicine, Hospital de Clínicas de Porto Alegre, Universidade Federal do Rio Grande do Sul, Porto Alegre/RS, Brazil; 3 Generar, Porto Alegre, Brazil

**Keywords:** ovary, ovarian reserve, fertility preservation, tissue culture techniques, gender reassignment surgery, ovário, reserva ovariana, preservação da fertilidade, técnicas de cultura de tecidos, cirurgia de redesignação sexual

## Abstract

**Objective** To evaluate the reproductive and histological characteristics of fresh cultured ovarian tissue from transgender male patients.

**Methods** An in vitro pilot study in which samples were collected during sex reassignment surgery for transgender male patients. The ovarian cortex was cut into fragments of 2 mm, 3 mm, and 4 mm, and placed in a 96-well plate suitable for cultivation at days 0, 2, 4, 6, and 8, when the histology was analyzed.

**Results** Stromal hyperplasia was observed in all samples, and it was not associated with the obtainment of primordial or primary follicles. Peripheral reduction in cell count was also a recurrent finding. Primordial and primary follicles were identified with a heterogeneous pattern in fragments from the same patient and from different patients, and follicles in more advanced stages of development (secondary and antral) were not found. There was an association between the diameter of the ovarian fragments and the identification of primary follicles ( *p* = 0.036). The number of days in culture was associated with histological signs of tissue damaging in the fragments ( *p* = 0.002). The total number of follicles identified in the samples with 2 mm in diameter was significantly lower than in those that measured 4 mm in diameter ( *p* = 0.031).

**Conclusion** A diameter of 4 mm is suitable for ovarian tissue culture with the benefit of ease of handling. Even after prolonged exposure to testosterone, the ovarian fragments presented primordial and primary follicles, maintaining viability throughout the days they were exposed to the culture. Freezing the ovarian cortex of transgender patients who will undergo surgery for gender reassignment would be an interesting option, in the future, for the preservation of fertility.

## Resumo

**Objetivo** Avaliar as características reprodutivas e histológicas de tecido ovariano cultivado a fresco de pacientes transexuais masculinos.

**Métodos** Estudo experimental *in vitro* e piloto, no qual amostras foram coletadas durante a cirurgia de redesignação de sexo para pacientes transexuais masculinos. O córtex ovariano foi cortado em fragmentos de 2 mm, 3 mm, e 4 mm, e colocado em placa de 96 poços própria para cultivo nos dias 0, 2, 4, 6 e 8, quando a histologia foi analisada.

**Resultados** Hiperplasia estromal foi observada em todas as amostras, e não esteve associada à obtenção de folículos primordiais ou primários. A redução periférica no número de células também foi um achado recorrente. Folículos primordiais e primários foram identificados com padrão heterogêneo em fragmentos do mesmo paciente e em fragmentos de pacientes diferentes, não sendo encontrados folículos em estágios mais avançados de desenvolvimento (secundários e antrais). Houve associação entre o diâmetro dos fragmentos ovarianos e a identificação dos folículos primários ( *p* = 0,036). O número de dias de cultura esteve associado a sinais histológicos de lesão tecidual nos fragmentos ( *p* = 0,002). O número total de folículos identificados nas amostras de 2 mm de diâmetro foi significativamente menor do que nas de 4 mm de diâmetro ( *p* = 0,031).

**Conclusão** O diâmetro de 4 mm parece ser mais adequado para a cultura de tecido ovariano com a vantagem de fácil manejo. Mesmo após exposição prolongada à testosterona, os fragmentos ovarianos apresentavam folículos primordiais e primários, e manteve a viabilidade ao longo dos dias de exposição à cultura. No futuro, o congelamento da cortical do ovário de pacientes transgêneros que se submeterão à cirurgia de redesignação sexual poderia ser uma opção interessante para a preservação da fertilidade.

## Introduction

At birth, the ovaries have a defined number of primordial follicles, which constitute the ovarian reserve. The activation of these follicles is responsible for the irreversible decline in reproductive function throughout the years, 1 and the rate of this reduction is variable among women. 2

One of the major causes of potentially-avoidable ovarian-reserve reduction is the use of gonadotoxic drugs in the treatment of neoplasms. These drugs can trigger exacerbated follicular activation/inhibition, leading to accelerated and premature depletion of the follicular reserve. Chemotherapeutic agents may also cause oocyte DNA damage. Understanding these mechanisms of oocyte-DNA repair seems crucial to comprehend the factors involved in maintaining and reducing the follicle pool throughout reproductive age and in procedures that may lead to ovarian injury. 3 Thus, preventing ovarian dysfunction induced by chemotherapy would be important to preserve the possibilities of natural or medically-assisted conception after treatments. 4

The methods currently used to preserve the fertility of patients undergoing cytotoxic and hormonal treatments are the cryopreservation of embryos, oocytes, and ovarian tissue. However, the available techniques have an indication limited to the patient's age and clinical status. 5 6

The development of culture conditions for immature germ cells is one of the biggest challenges in reproductive medicine, with the goal of obtaining competent oocytes. Complete in vitro growth of primordial follicles with subsequent in vitro fertilization, followed by live-embryo transfer, has been achieved, so far, only in mice. 7

In view of the complex regulatory system of follicular development, the challenge remains to create increasingly elaborate and complex culture systems to promote an environment similar to that of the ovary in vivo, and thus, through cryopreserved ovarian-tissue fragments, support follicular growth. The impossibility of access to human ovarian tissue, free of diseases, of young women – due to ethical aspects – results in the difficulty of establishing an efficient in vitro ovarian culture.

Gender reassignment surgery for transgender male individuals, which includes the excision of the ovaries, allows the gonads to be used for study purposes. The testosterone therapy to which these patients are submitted for long periods leads to reversible amenorrhea, with preserved ovarian follicles, without depleting or affecting the development of primordial follicles. 8

Therefore, due to the limited amount of human tissue available for clinical use and research, as well as the reduced numbers of follicles in samples obtained from older patients or from those with ovarian disease, the ovaries of transgender male patients are an opportunity to study this noble organ in research. The aim of the present study was to evaluate the reproductive and histological characteristics of fresh cultured ovarian tissue from transgender male patients.

## Methods

An experimental in vitro pilot study was performed. The experiments were performed in the Endocrine and Tumoral Molecular Biology Laboratories of the Department of Physiology at Universidade Federal do Rio Grande do Sul (UFRGS), in the city of Porto Alegre, Southern Brazil, and in Experimental Pathology Unit of the Experimental Research Center at Hospital de Clínicas de Porto Alegre (HCPA). This project was supported by Fundo de Incentivo à Pesquisa e Eventos-HCPA (FIPE-HCPA, in Portuguese), under #2018–0462.

The samples were collected from the ovarian cortex of patients from the Gender Identity Program (Programa de Identidade de Gênero, PROTIG, in Portuguese) at HCPA who underwent gender reassignment surgery with an indication not related to the present study. The criteria for inclusion were patients who underwent pan-hysterectomy, aged between 20 and 45 years, who did not present ovarian neoplasia. The sample size was determined by convenience.

After collecting fragments from the ovarian cortex, the specimens were sent for an anatomopathological analysis. They were identified and transported under refrigeration to the laboratory in a medium composed of Hank salt solution (Gibco BRL, Grand Island, NY, United States) and 1% kanamycin. In a laminar-flow hood, excess blood was removed, and the samples were cut into fragments of 2 mm, 3 mm, and 4 mm in diameter with a disposable biopsy punch (Kolplast, Itupeva, SP, Brazil). After cutting, the samples were plated.

The ovarian-cortex fragments were placed in a 96-well plate. Each well was filled with 200 μL of Dulbecco's Modified Eagle Medium (DMEM, Gibco BRL) supplemented with 1% antibiotic (streptomycin), 25 mIU/mL of recombinant follicle-stimulating hormone (FSH) (GONAL-f, Merck, Darmstadt, Germany) and 5% fetal bovine serum (FBS, Gibco BRL). The tissue fragments were grown at 37°C in a humidified incubator, with a constant injection of 5% CO _2_ for 2, 4, 6, and 8 days. The culture medium was changed every 48 hours, and the fragments were observed everyday under an inverted microscope. After 2, 4, 6, or 8 days of culture, the fragments were fixed in 10% buffered formaldehyde.

The formaldehyde-fixed samples were sent to the HCPA Experimental Pathology Unit, where they were embedded in paraffin and cut into a series of 5 µm sections for the histological analysis. The slides were stained with hematoxylin & eosin.

To limit the effect of heterogeneous follicular distribution within the samples of the ovarian cortex submitted to culture, random sections were extracted from each piece of ovary, with the purpose of covering the entire fragment. The slides were evaluated under an optical microscope by an experienced pathologist.

A primordial follicle was defined as the presence of an oocyte surrounded by a layer of spindle-shaped granulosa cells and a primary follicle is an oocyte surrounded by cuboidal granulosa cells. Secondary follicles are characterized by an oocyte that is completely surrounded by a zona pellucida and the presence of at least two layers of granulosa cells. Antral follicles are defined by the presence of an antral cavity. 9

The data were entered twice, revised and analyzed using the Statistical Package for the Social Sciences PASW Statistics for Windows, SPSS Inc., Chicago, IL, United States) software, version 18.0. The qualitative variables were expressed as absolute (n) and relative (n%) frequencies. The Fisher exact test was applied, and Yates correction for continuity was used when indicated. The quantitative variables were expressed as medians, as distributed by the Shapiro-Wilk normality test; so, the Kruskal-Wallis test and Dunn-Bonferroni post hoc test were applied. For all analyzes, the significance level was set at 5%.

In order to obtain authorization to use the ovarian fragments in the present study, patients were informed about the research and invited to participate by signing the informed consent form (ICF), knowing that they could withdraw at any time.

After the experiments, the residues were packed in white bags, closed, sealed, and identified with a biological residue label with all the required information, and delivered to the competent collection service of the institution. Phenol residues were treated as chemical waste and collected by the collection service based at the Chemistry Institute of UFRGS.

## Results

The ovarian fragments studied were obtained at the time of the gender reassignment surgery (pan-hysterectomy). The age of the patients ranged from 25 to 34 years, and all of tehm had used testosterone before surgery. The age in which they started using the hormone, the duration and time between discontinuation and surgery are shown in Table 1 . In the macroscopic analysis, 3 of the 4 ovaries had cystic follicles.

**Table 1:** Characteristics of the patients

	Patient 1	Patient 2	Patient 3	Patient 4
Age (years)	34	25	27	32
Age at the beginning of the treatment (years)	31	22	24	29
Duration of use (months)	27	30	22	33
Presurgical hormone discontinuation (months)	1	2	12	3

**Fig. 1:**
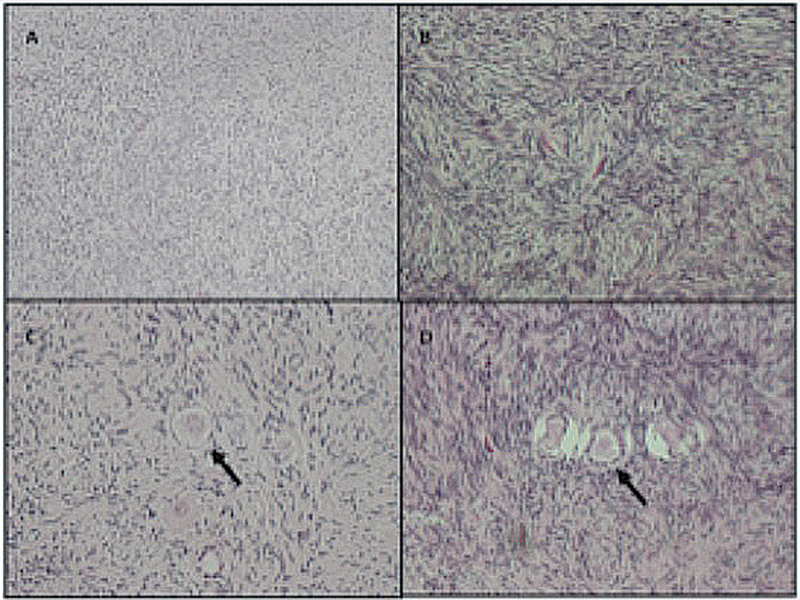
Histological sections of ovaries stained with hematoxylin & eosin (H&E), showing ( **A** ) stromal hyperplasia (4x); ( **B** ) stromal hyperplasia with the magnification of the objective lens (40x); ( **C** ) primordial follicles and ( **D** ) primary follicles (40x).

The histology of the ovarian fragments was analyzed at days 0, 2, 4, 6, and 8 of culture. Stromal hyperplasia was observed in all samples, regardless of the culture day ( Fig. 1 ). Peripheral reduction in the cell count was also a recurrent finding, related to the advancing days of culture. Primordial and primary follicles were identified with a heterogeneous distribution pattern in fragments from the same patient and in those of different patients, and follicles in more advanced stages of development (secondary and antral) were not found. In all the ovarian-cortex fragments analyzed, the total number of primordial and primary follicles identified was 267 and 224 respectively.

The total number of follicles found per patient according to the culture day is shown in Table 2 . There was no association between the culture day and the number of follicles found.

**Table 2:** Total number of follicles per patient according to the culture day (samples in triplicates)

	Number of follicles				
	Patient 1	Patient 2	Patient 3	Patient 4	Total
Day 0	18	4	14	36	72
Day 2	–	19	4	45	68
Day 4	40	8	13	132	193
Day 6	12	4	13	74	103
Day 8	13	–	5	37	55
Total	83	35	49	324	491

There was an association between the diameter of the ovarian fragments and the identification of primary follicles. Fragments with a diameter ≥ 3 mm showed significantly more primary follicles ( *p* = 0.036), which was not observed regarding primordial follicles ( Table 3 ).

**Table 3:** Association between the diameter of the tissue fragments and the presence of primordial and primary follicles

	Tissue diameter		*p* -value *
	≤ 2 mm	≥ 3 mm	
Primordial follicle			
No	7 (46.7%)	13 (39.4%)	0.755*
Yes	8 (53.3%)	20 (60.6%)	
Primary follicle			
No	9 (60%)	8 (24.2%)	0.036 ^#^
Yes	6 (40%)	25 (75.8%)	

Notes: *Fisher exact test. ^#^ Yates correction for continuity. Statistical significance accepted when *p* -value ≤ 0.05.

**Fig. 2:**
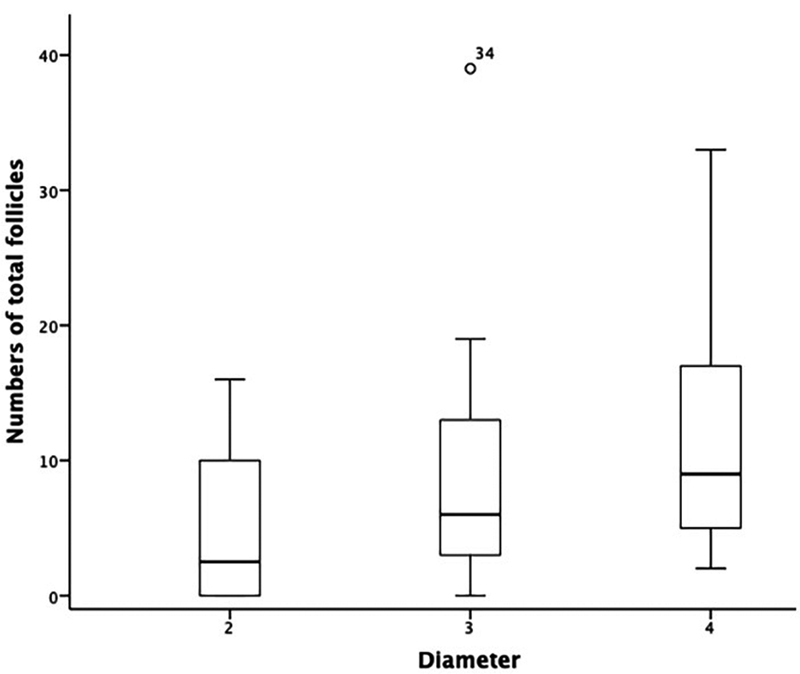
Total number of follicles in samples with 2 mm, 3 mm, and 4 mm in diameter. Note: *2 mm different from 4 mm.

The presence of stromal hyperplasia was not associated with the obtainment of primordial or primary follicles ( *p* = 0.042) ( Table 4 ), neither were the number of days in culture ( Table 5 ). However, the time in the culture was associated with histological signs of tissue damaging in the fragments ( *p* = 0.002) ( Table 6 ). The total number of follicles identified in the samoples with 2 mm in diameter was significantly lower than in those with 4 mm in diameter ( *p* = 0.031; Kruskal-Wallis test, Dunn-Bonferroni post hoc test, and the normality was tested with the Shapiro-Wilk test). However, when comparing the samples with a diameter of 3 mm with those with 2 mm or 4 mm, no difference was found ( Fig. 2 ).

**Table 4:** Association between primordial and primary follicles and stromal hyperplasia

	Stromal hyperplasia		*p* -value *
	No	Yes	
Primordial follicle			
No	12 (41.4%)	5 (31.2%)	0.541*
Yes	17 (58.6%)	11 (68.8%)	
Primary follicle			
No	6 (20.7%)	8 (50%)	0.090 ^#^
Yes	23 (79.3)	8 (50%)	

Notes: *Fisher exact test. ^#^ Yates correction for continuity. Statistical significance accepted when *p* -value ≤ 0.05.

**Table 5:** Association between the presence of primordial and primary follicles and the number of days in culture (≤ 4 days or ≥ 6 days)

	Number of days of culture		*p* -value *
	≤ 4 days	≥ 6 days	
Primordial follicle			
No	13 (44.8%)	7 (36.8%)	0.803 ^#^
Yes	16 (55.2%)	12 (63.2%)	
Primary follicle			
No	8 (27.6%)	9 (47.4%)	0.274 ^#^
Yes	21 (72.4%)	10 (52.6%)	

Notes: ^#^ Yates correction for continuity. Statistical significance accepted when *p* -value ≤ 0.05.

**Table 6:** Association between culture time (≤ 4 days and ≥ 6 days) and histological signs of tissue damaging

	Number of days of culture		*p* -value *
	≤ 4 days	≥ 6 days	
Tissue damaging			
No	20 (74.1%)	5 (26.3%)	0.002*
Yes	7 (25.9%)	14 (73.7%)	

Notes: *Fisher exact test. Statistical significance accepted when *p* -value ≤ 0.05.

## Discussion

The establishment of in vitro techniques for the culture of human ovarian tissue would enable a better understanding of the mechanisms involved in follicular growth and, consequently, the expansion of ovarian-preservation methods. Ethical issues minimize access to human ovarian tissue for research, with a consequent difficulty in standardizing and developing protocols to cultivate this type of tissue. Transgender male patients, at the time of the gender reassignment surgery, which includes the excision of the ovaries, make it possible to obtain young human ovarian tissue without pathologies.

All patients submitted to this surgery undergo hormonal therapy with testosterone, as part of the treatment of adaptation to the identified gender. In the present study, even after prolonged exposure to testosterone, the ovaries had viable primordial and primary follicles, maintaining viability throughout the days they were exposed to the culture. Van Den Broecke et al. (2001) 8 proposed that the ovarian cortex of these patients could be used experimentally to obtain primary follicles, and, from these, in xenograft models (mice), to reach later stages of growth. However, the authors 8 were not able to obtain antral follicles.

In our study, similarly as reported by Schmidt et al. while analyzing the cortex of three human ovaries (2003) 10 , a heterogeneous distribution of follicles was found in the human ovarian cortex. The primordial follicles showed a density variation of more than two orders of magnitude in random pieces of cortical tissue from the same ovary, and the developmental stage of the follicles had a heterogeneous distribution. 10 Another important finding to be highlighted is that fragments of the ovarian cortex measuring 4 mm in diameter enable the identification of a larger number of follicles than smaller sections, and they are also easier to manipulate during plate cultivation, with no differences regarding tissue damaging. It was also possible to identify peripheral hypocellularity, which was observed as the days in the culture advanced, which is characteristic of tissue culture, in which cells expand around the fragment and start to adhere to the material in the culture plate. It seems logical that bigger samples would present more follicles. In larger fragments, intraovarian autocrine and paracrine factors may be more available, playing a significant role in follicular density. 10

Stromal hyperplasia, a recurrent observation in the evaluation of fragments, has been described in studies 11 12 13 14 that evaluated the ovarian tissue of patients with a history of hormone therapy with testosterone. These authors 11 12 13 14 relate this finding to the androgenic microenvironment resulting from previous exposure to testosterone, and this association is due to the fact that this process is frequently observed in the ovaries of patients with polycystic ovary syndrome (PCOS), with the androgenic microenvironment being one of the pathophysiological basis of this disease.

The presence of cystic follicles on macroscopy identified in the present study is described in the literature in patients with PCOS. A study 15 that evaluated the ovarian histology of 12 transgender male patients described enlarged and multifollicular ovaries, a consequence of the direct or indirect effect of androgens on the proliferation and growth of stromal ovarian cells. The follicular morphological changes found were attributed to the increased stimuli of growth factors exerted by androgens. 15

Comparative studies, such as the one by Pache et al. (1991), 16 which included 29 ovaries obtained from 17 transgender patients in amenorrhea after androgenic hormone therapy and 14 control ovaries, showed greater ovarian volume, collagen thickening of the cortex, and stromal hyperplasia accompanied by clusters of stromal cells in the ovaries of transgender patients. Another comparative study 17 included ovaries of 19 transgender patients and 12 control patients and found, in the ovaries of transgender patients, an increase in volume, multiple cystic follicles (89.5%), diffuse ovarian stromal hyperplasia (84.2%), collagenization of the external cortex (68.4%), and luteinization of stromal cells (26.3%). 17

Obtaining follicles in more advanced stages of development was not possible, nor was there any development of follicles in vitro, probably due to inhibition by testosterone, despite what was previously shown by Van Den Broecke et al. (2001). 8 It has been shown that gender reassignment surgery is a unique opportunity to obtain human ovaries for research, with proven follicular and tissue viability in up to 8 days of culture. Further studies aiming at developing cortex culture protocols capable of supporting follicular survival, growth, and germ-cell maturation in humans are essential.

## Conclusion

The present study provides some reflections on the perspectives in the field of human ovary research. In vitro ovarian tissue culture, in addition to being a simpler option than follicular culture, also has the potential of elucidating the complex pathways of follicular activation and suppression. The 4-mm diameter is suitable for ovarian tissue culture, with the benefit of ease of handling compared with the diameters of 2 mm and 3 mm, with no difference in tissue damage. Freezing of the ovarian cortex of transgender patients who will undergo surgery for gender reassignment would be an interesting option, in the future, for the preservation of fertility. The possibility of obtaining viable follicles, even after using hormone therapy, makes the first step on this path feasible.
